# OsHsfB4d Binds the Promoter and Regulates the Expression of *OsHsp18.0-CI* to Resistant Against *Xanthomonas Oryzae*

**DOI:** 10.1186/s12284-020-00388-2

**Published:** 2020-05-27

**Authors:** Wei Yang, Yanhu Ju, Liping Zuo, Luyue Shang, Xinru Li, Xiaoming Li, Shangzong Feng, Xinhua Ding, Zhaohui Chu

**Affiliations:** 1 State Key Laboratory of Crop Biology, Shandong Agricultural University, Tai’ an, 271018 Shandong PR China; 2 Shandong Pengbo Biotechnology Co LTD, Tai’ an, 271025 Shandong PR China; 3 College of Agronomy, Shandong Agricultural University, Tai’ an, 271018 Shandong PR China; 4 Shandong Provincial Key Laboratory for Biology of Vegetable Diseases and Insect Pests, College of Plant Protection, Shandong Agricultural University, Tai’ an, 271018 Shandong PR China; 5 Agro-technical Popularization Centre of Linyi City, Linyi, 276000 Shandong PR China

**Keywords:** Defense response, Heat shock factor, Heat shock protein, *Oryzae sativa*, *Xanthomonas oryzae*

## Abstract

**Background:**

Bacterial leaf streak (BLS) and bacterial blight (BB) are two major prevalent and devastating rice bacterial diseases caused by the Gram-negative bacteria of *Xanthomonas oryzae* pv. *oryzicola* (Xoc) and *Xanthomonas oryzae* pv. *oryzae* (Xoo), respectively. Previously, we identified a defence-related (*DR*) gene encoding a small heat shock protein, OsHsp18.0-CI, that positively regulates BLS and BB resistance in rice.

**Results:**

To reveal the regulatory mechanism of the *OsHsp18.0*-*CI* response to Xoc and Xoo, we characterized the class B heat shock factor (Hsf), OsHsfB4d, through transcriptional analysis and a transgenic study. *OsHsfB4d* is upregulated post inoculation by either the Xoc strain RS105 or Xoo strain PXO99a in Zhonghua 11 (wild type, ZH11) as well as in *OsHsp18.0*-*CI* overexpressing rice plants*.* Transient expression of *OsHsfB4d* can activate the expression of green fluorescent protein (GFP) and luciferase (Luc) via the *OsHsp18.0-CI* promoter. Rice plants overexpressing *OsHsfB4d* exhibited enhanced resistance to RS105 and PXO99a as well as increased expression of *OsHsp18.0*-*CI* and pathogenesis-related genes. Furthermore, we found that OsHsfB4d directly binds to a DNA fragment carrying the only perfect heat shock element (HSE) in the promoter of *OsHsp18.0-CI*.

**Conclusion:**

Overall, we reveal that OsHsfB4d, a class B Hsf, acts as a positive regulator of *OsHsp18.0-CI* to mediate BLS and BB resistance in rice.

## Background

Rice is an important staple crop worldwide that represents 40% of total grain output and nearly 60% of global food consumption (Sharma et al. 2012). However, it has been shown to suffer more than 70 diseases caused by fungi, bacteria, viruses and nematodes during rice growth (Niño-Liu et al. 2006; Ke et al. 2017). There are two major bacterial diseases, bacterial blight (BB) and bacterial leaf streak (BLS), caused by the gram-negative bacteria *Xanthomonas oryzae* that frequently occur in rice. BB is caused by *Xanthomonas oryzae* pv. *oryzae*, which enters into the rice leaf through hydathodes or wounds and colonizes in the xylem vessels. But BLS is caused by *Xanthomonas oryzae* pv. *oryzicola* (Xoc), which penetrates into the leaf through stomata or wounds and colonizes the intercellular space of leaf tissue and finally results in water-soaked stripe lesions (Niño-Liu et al. 2006; Ke et al. 2017; Ju et al. 2017). Currently, BB is well studied for host resistance. Over 40 major resistance genes and 30 defense-related (DR) genes were identified to control the race-specific or spectrum resistance to Xoo isolates (Ke et al. 2017; Ju et al. 2017). BLS is becoming a major concern due to its high prevalence and seriously affecting the yield and quality of rice production (Xu et al. 2010; Zhang et al. 2015). To date, only the *Xo1* locus, which encodes a putative receptor, has been reported to confer qualitative resistance against the African clade of Xoc strains but not Asia strains (Triplett et al. 2016). Otherwise, BLS resistance was considered to be controlled by a number of quantitative trait loci (QTLs), such as *qBlsr5a*, which may consist of *xa5*, *OsPGIP1* and *OsPGIP4* from the rice variety Acc8558 (Xie et al. 2014; Feng et al. 2016; Wu et al. 2019).

Several *DR* genes have been demonstrated to exhibit upregulated expression upon Xoc inoculation and to positively or negatively regulate the rice BLS resistance (Xu et al. 2013). Overexpression of the mitogen-activated protein kinase gene *OsMPK6* increased susceptibility to Xoc strain RS105, implying the negative regulation of rice BLS resistance (Shen et al. 2010). Consistent with the negative regulation of rice immunity, suppression of *DR* genes, such as *OsWRKY45–1* (Tao et al. 2009), *OsDEPG1* (Guo et al. 2012), *OsNRRB* (Guo et al. 2014), *OsImpα1a* and *OsImpα1b* (Hui et al. 2019), could enhance the resistance to Xoc. In contrast, other *DR* genes, including *OsGH3–2* (Fu et al. 2011), *OsPGIP4* (Feng et al. 2016), *RGA4*/*RGA5* (Hutin et al. 2016), *OsHsp18.0-CI* (Ju et al. 2017), *XCRK* (Zhang et al. 2017), *MPKK10.2* (Ma et al. 2017), *OsPSKR1* (Yang et al. 2019) and *OsPGIP1* (Wu et al. 2019), may positively regulate BLS resistance in rice. However, constitutively expressed of *DR* genes often result in enhanced resistance as well as impairment of agronomic traits such as yield and quality (Wiesner-Hanks et al. 2018). Therefore, understanding the transcriptional regulation mechanisms of *DR* genes and fine-tuning their regulation ability is critical and ideal for breeding resistant rice varieties.

Heat shock proteins (Hsps) are conserved across a wide diversity of organisms. They are chaperones that assist in protein folding and prevent irreversible protein aggregation (Waters 2012). Commonly, the transcription of a heat shock protein gene is regulated by heat shock factors (Hsfs) in plants. Plants contain multimember Hsf gene families organized into evolutionarily conserved and structurally distinct A, B and C classes (Kotak et al. 2004; Scharf et al. 2012). The modular Hsf structure comprises an N-terminal DNA binding domain (DBD), an adjacent oligomerization domain (OD or HR-A/B region), nuclear localization and export signals (NLS/NES) and a C-terminal activation domain (CTAD) (Lavania et al. 2018). Class A Hsfs specifically contain a unique C-terminal activation domain with aromatic hydrophobic acidic (AHA) motifs, while class B and class C Hsfs are characterized by the lack of an activation domain (Nover et al. 2001; Baniwal et al. 2004). Normally, Hsfs activates or inhibits the expression of the targeted Hsp gene by directly binding to the heat shock element (HSE), a consensus sequence of 5′-nGAAn-3′ embedded in the promoter (Sakurai and Enoki 2010; Scharf et al. 2012). Class A Hsfs are generally considered positive regulators, while class B and C Hsfs are considered negative regulators.

Currently, the role of Hsfs in basal tolerance to heat and other abiotic stresses is well established in plants (Hahn et al. 2011; Scharf et al. 2012). However, the role of Hsfs in plant disease resistance is poorly understood. Overexpression of *AtHsfA1b* resulted in increased resistance to *Pseudomonas syringae* (*Pst*) DC3000 and *Hyaloperonospora Arabidopsis* pv. WACO9 (Hpa) in *Arabidopsis* (Bechtold et al. 2013). *SlHsfA1a* positively regulated *Wfi1*, an *RBOH* gene that triggers the production of reactive oxygen species (ROS) in the apoplast during *Meloidogyne incognita* infection of the roots of tomato plants (Zhou et al. 2018). In contrast, class B Hsf members play a negative regulatory role in disease resistance mechanisms. *AtHsfB1* and *AtHsfB2b* negatively regulate the expression of the plant defensin genes *PDF1.2a* and *b* (Kumar et al. 2009). As a result, the single mutant *AtHsfB2b* and the double mutant *AtHsfB1/AtHsfB2b* exhibited resistance to the necrotrophic fungus *Alternaria brassicicola* (Liu and Charng 2013). In addition, overexpressing the grape *VpHsf1* gene (encoding a member of the B family) in tomatoes can increase the sensitivity of plants to *Erysiphe necator* (Peng et al. 2013).

Rice contains a total of 25 typical Hsfs members in its genome. Among them, 22, 10 and 14 *OsHsfs* have been identified that respond to heat, chilling and oxidative stress, respectively (Mittal et al. 2009). However, the functions of most OsHsfs have not been revealed. Previously, we identified a small heat shock protein encoded by *OsHsp18.0-CI* that positively regulates rice BLS and BB resistance (Ju et al. 2017; Zuo et al. 2019). Using an RNA-seq strategy, a number of differentially expressed genes (DEGs) involved in basal defence were shown to be enriched in *OsHsp18.0-CI*-overexpressing (OE) lines compared with wild-type ZH11 (Ju et al. 2017; Zuo et al. 2019). Among the characterized DEGs, one of the candidate Hsfs, *OsHsfB4d*, was identified by deeply analysing the comparative transcriptome data and investigating the regulatory interaction with *OsHsp18.0-CI*. Importantly, we found that *OsHsfB4d* acts as a positive regulator to activate the expression of *OsHsp18.0-CI* by directly targeting its promoter sequence.

## Results

### The Expression of OsHsfB4d is Induced by Xanthomonas Oryzae pv. Oryzae and X. Oryzae pv. Oryzicola

**Fig. 1:**
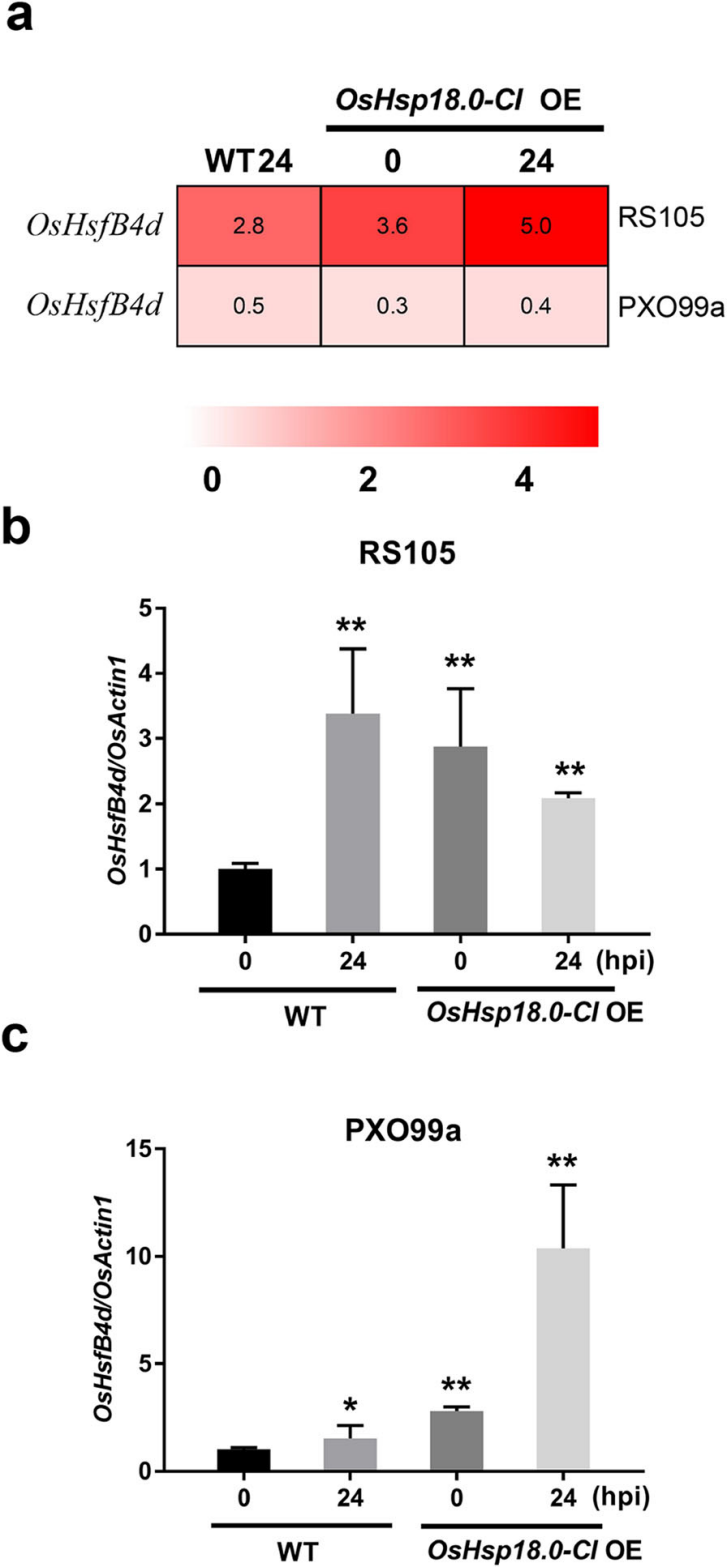
*OsHsfB4d* expression is induced by *Xanthomonas oryzae* pv. *oryzicola* (Xoc) and *Xanthomonas oryzae* pv*. oryzae* (Xoo) inoculation. **a** Hierarchical cluster analysis of *OsHsfB4d* using the *OsHsp18.0-CI* RNA-seq data. Quantification of the expression level of *OsHsfB4d* in response to RS105 **(b)** or PXO99a **(c)** by qRT-PCR. Fully expanded leaves of six-week-old ZH11 and *OsHsp18.0-CI*-OE plants were harvested at 0 and 24 h post inoculation. The bars represent the mean (three replicates for gene expression) ± SD. The significance of expression compared to that at 0 h in the WT at *P* values of less than 0.05 and 0.01 are indicated by “*” and “**”, respectively

As Hsfs specifically regulate the expression of Hsp genes, to reveal the regulatory mechanism of *OsHsp18.0-CI* in rice immunity, we analysed the transcriptome data from rice lines overexpressing *OsHsp18.0-CI* cDNA under the control of the *Ubi* promoter post Xoc or Xoo inoculation (Ju et al. 2017; Zuo et al. 2019). As shown in the supporting file Figure S1, we investigated the transcriptional data for all class A and B Hsfs. Several *Hsfs* were upregulated in transgenic plants or by pathogen inoculation, including *OsHsfA2f*, *OsHsfA7*, *OsHsfB1* and *OsHsfB4d*. *OsHsfB4d* attracted attention because its expression was significantly induced by both the Xoc and Xoo strains. The transcription level of *OsHsfB4d* was approximately 8-fold increased compared with that in the control 24 h post inoculation (hpi) with Xoc strain RS105. *OsHsfB4d* was also significantly upregulated in *OsHsp18.0-CI* OE plants compared with wild-type ZH11 plants without inoculation with RS105 (Fig. 1a). Upregulation pattern for *OsHsfB4d* was also identified in response to the Xoo strain PXO99 (Fig. 1a). The results of the RNA-seq data analysis were further validated by qRT-PCR to confirm the induction of *OsHsfB4d* expression during Xoc and Xoo inoculation (Fig. 1b, c). The above results demonstrated that increased level of transcripts of *OsHsfB4d* was identified in ZH11 rice after the two bacterial pathogens challenge, suggesting that *OsHsfB4d* may be involved in the resistance of rice to these two pathogens.

### OsHsfB4d Transiently Activated the Expression of *OsHsp18.0-CI*

**Fig. 2:**
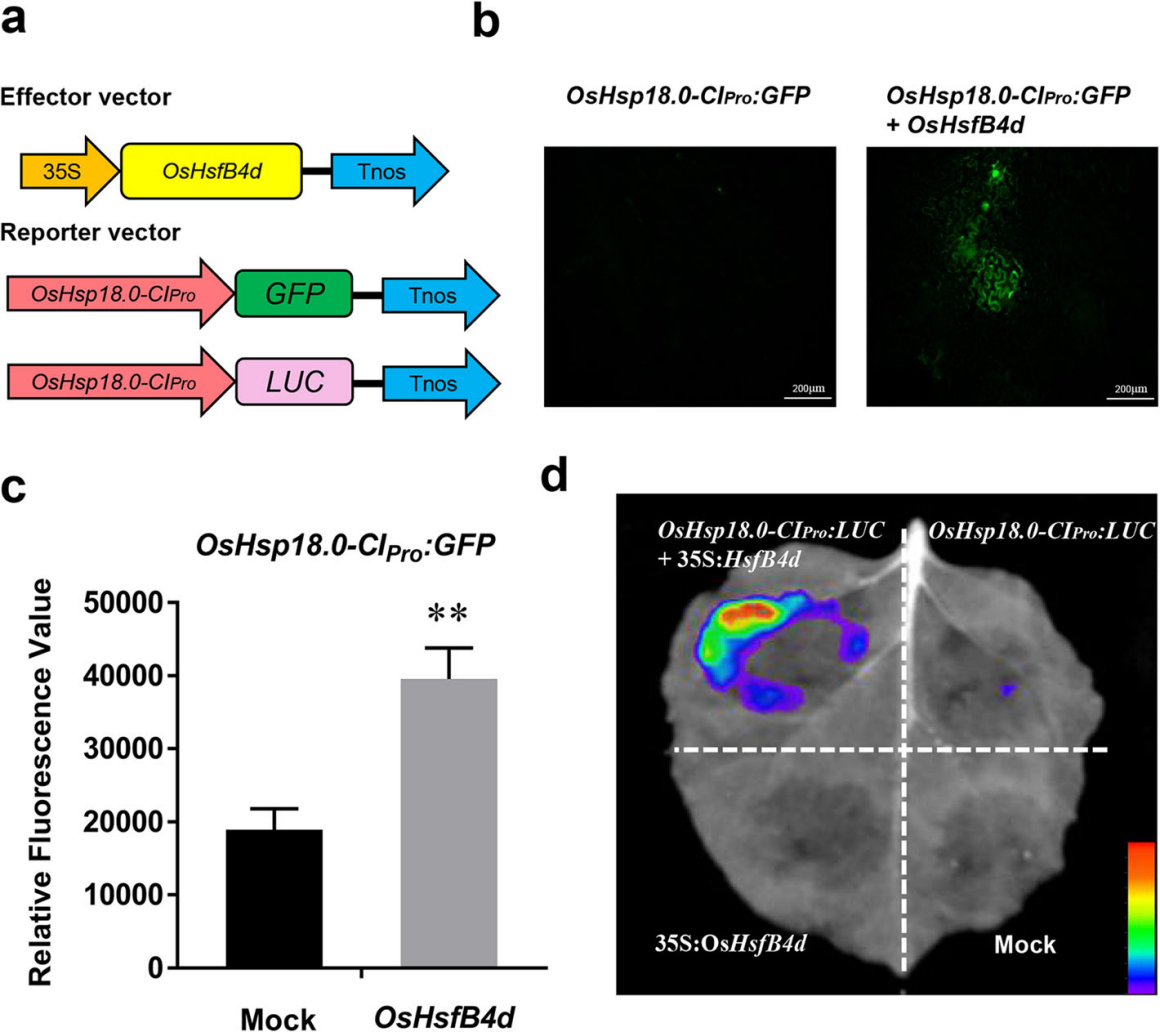
Fluorometric assays of GFP or LUC driven by *OsHsp18.0-CI* promoter constructs. **a** Diagram of the constructs of OsHsfB4d and the *OsHsp18.0-CI* promoter. **b** GFP fluorescence of the *OsHsp18.0-CI* promoter DNA construct (OsHsp18.0-CI_Pro_:GFP). Coexpression with or without the OsHsfB4d in *N. benthamiana*. Images were photographed at 2 d after infiltration. Bar = 200 μm. **c** Quantitative analysis of the fluorescence intensity. The results are presented as the average fluorescence intensity using ZEN software. The experiments were repeated three times. ** indicates significant differences according to Fisher’s least-significant difference test (*P* < 0.01). **d** Luciferase assay of the co-expressed OsHsfB4d and the promoters of *OsHsp18.0-CI* (OsHsp18.0-CI_Pro_:LUC). Mock represents the 10 mM MgCl_2_ solution

To evaluate whether OsHsfB4d could regulate the expression of *OsHsp18.0-CI*, a transient expression assay was performed by coexpressing *OsHsfB4d* and green fluorescent protein (GFP) or luciferase (LUC) by using *OsHsp18.0-CI* promoter. As shown in Fig. 2a, different combinations of constructs were transiently introduced into *Nicotiana benthamiana* leaves by *Agrobacterium tumefaciens*-mediated transformation, and GFP activity was assayed 24 h after infiltration. No obvious fluorescence was detected in the control plant leaves, whereas significant GFP fluorescence was observed in *OsHsfB4d* plants with reporter constructs (Fig. 2b). The quantification of the GFP fluorescence intensity was performed to obtain a similar result (Fig. 2c). In addition, coexpression with *OsHsfB4d* also activated the fluorescence of the OsHsp18.0-CI_pro_:LUC construct (Fig. 2d). These results indicated that *OsHsfB4d* might act as a regulator and activate the expression of *OsHsp18.0-CI in planta.*

### OsHsfB4d-Overexpressing Lines Conferred Enhanced Disease Resistance to BLS and BB

**Fig. 3:**
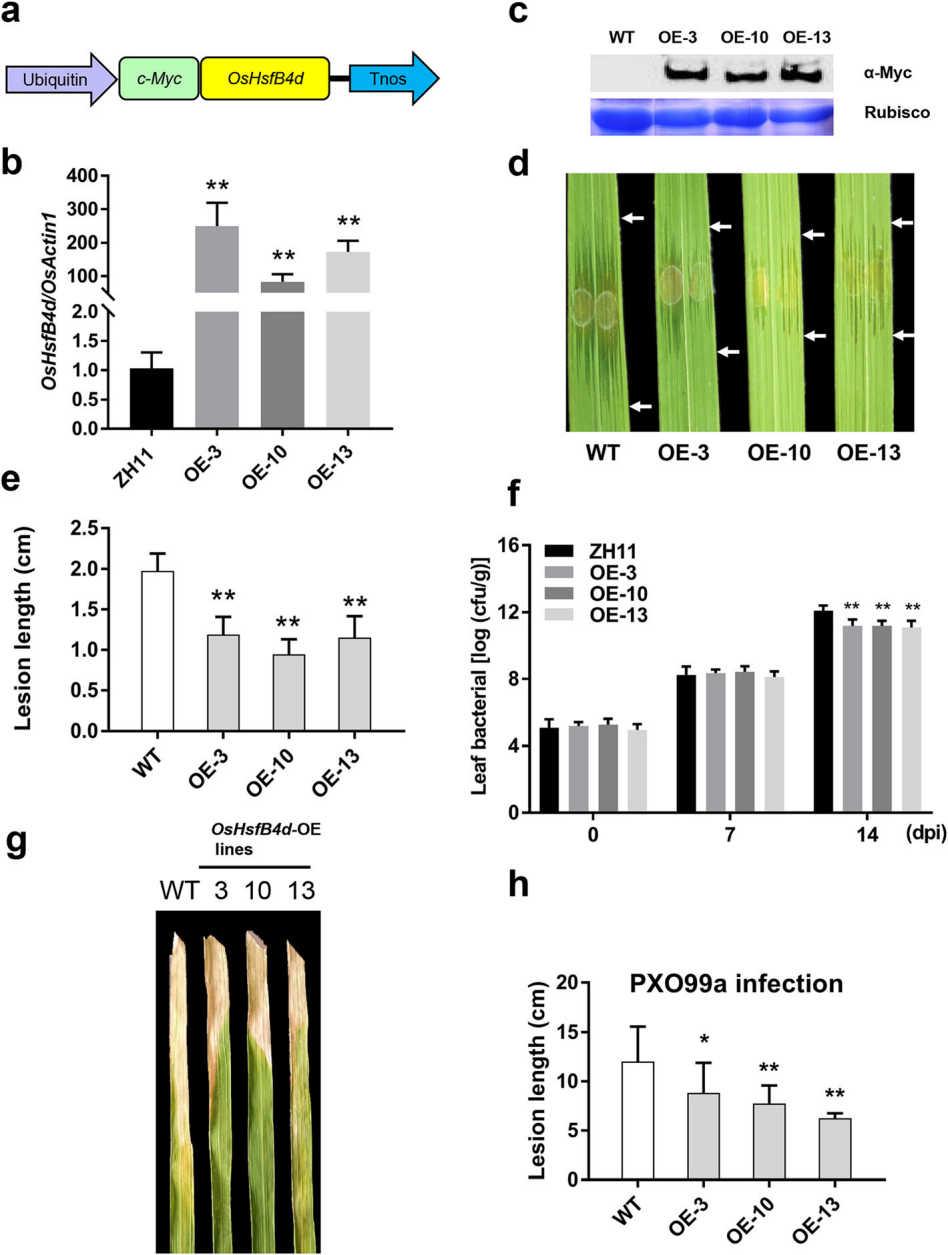
Resistance performance of transgenic *OsHsfB4d* OE plants from the T_2_ generation. **a** Diagram of the OsHsfB4d overexpression construct. **b** Relative expression levels of *OsHsfB4d* in three identified T_2_ transgenic lines. *OsActin* was used as an internal control. Bars represent the mean (three replicates for gene expression) ± SD. **c** Immunoblot analysis of OsHsfB4d in transgenic lines. Total protein extracts were separated by SDS-polyacrylamide gel electrophoresis (SDS-PAGE) and probed with antibodies against the *c*-Myc tag (α-Myc). The stained Rubisco protein shows that equal amounts of protein sample were loaded. The immunoblot band shows OsHsfB4d-Myc of approximately 35 kD. **d** Representative lesion sites in *OsHsfB4d* OE leaves at 14 dpi with Xoc strain RS105. **e** Lesion lengths scored for T_2_ transgenic plants with pCXUN::OsHsfB4d. Eight individuals were scored for each line and the WT. “**” indicates significant (*t* test, *P* < 0.01) differences (*n* = 16). **f** Bacterial population growth in leaves from *OsHsfB4d* OE and WT plants inoculated with RS105. Colonies were counted with the leaf segment up to 5 cm from the inoculation site. Error bars represent the standard deviation of three independent leaves. The bars represent the mean ± SD. “**” indicates significant (*t* test, *P* < 0.01) differences. **g** The phenotypes of the PXO99a lesions that developed on ZH11 and OsHsfB4d-OE lines at 14 dpi. **h** Lesion lengths were scored for OsHsfB4d-OE lines (*n* = 10). Asterisks indicate significant difference between transgenic and wildtype (WT) plants at **P < 0.01 or *P < 0.05

To investigate the role of *OsHsfB4d* in the disease resistance response, an overexpression vector expressing *OsHsfB4d* (OE) was constructed and introduced into the rice variety ZH11 (Fig. 3a). From the T_2_ generation, three homozygous transgenic lines, OE3, OE10 and OE13, were selected for further investigation. The transcription level of *OsHsfB4d* was increased approximately 249.2-fold, 82.8-fold and 172.5-fold in OE3, OE10 and OE13 compared to that in the WT, respectively (Fig. 3b). The increased content of OsHsfB4d protein was also confirmed by a western blotting assay with a *c*-Myc-tagged antibody (Fig. 3c). The fully expanded leaves of six-week-old plants were used for disease assessment by inoculation with RS105. The lesion length in the WT was recorded as being 1.98 ± 0.21 cm on average. Compared to the WT, the three OE lines showed shorter average lesion lengths of 1.19 ± 0.22 cm, 0.94 ± 0.19 cm and 1.15 ± 0.27 cm for OE3, OE10 and OE13, respectively (Fig. 3d, e). Consistently, though there are no significant difference between WT and OE lines at 7 dpi, the bacterial populations in the leaves of the OE lines were significantly decreased compared to those in the WT at 14 dpi (Fig. 3f). Overall, these results suggested that overexpression of *OsHsfB4d* enhanced rice resistance to RS105.

We also investigated the BB resistance for the OE lines. As shown in Fig. 3g, the lesion lengths of all three OE lines for PXO99a at 14 dpi were measured as 8.83 ± 3.05 cm, 7.75 ± 1.81 cm and 6.25 ± 0.50 cm, respectively (Fig. 3h). Which showed more shorter lesion lengths compared with WT (12.00 ± 3.55 cm). Therefore, OsHsfB4d could be considered as a positive regulator involved in the response to Xoc and Xoo.

### Activated Expression of Pathogenesis-Related (*PR*) Genes in *OsHsfB4d* OE Lines

**Fig. 4:**
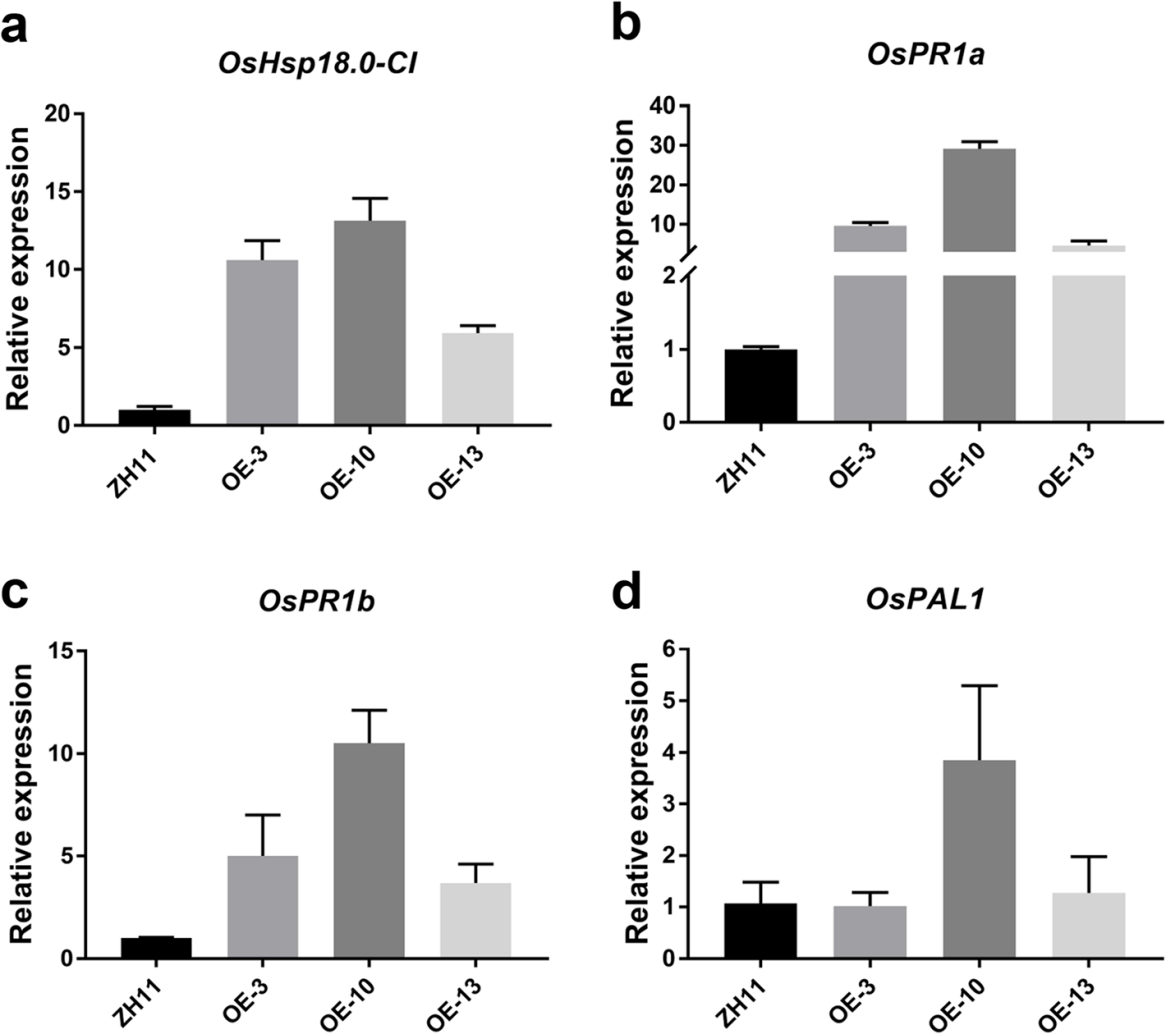
Overexpressing *OsHsfB4d* activates the expression of *OsHsp18.0-CI* and pathogenesis-related genes. Quantitative RT-PCR was performed for *OsHsp18.0-CI***(a)** and three *PR* genes, including *OsPR1a***(b)**, *OsPR1b***(c)** and *OsPAL1***(d)**, in 6-week-old wild-type and *OsHsfB4d* overexpression lines. The data are shown as the mean ± SD of three biological replicates. The experiments were repeated two times with similar results

As a candidate regulator of *OsHsp18.0-CI*, we first investigated the transcription level of *OsHsp18.0-CI* in rice lines OE3, OE10 and OE13. As shown in Fig. 4a, the relative expression of *OsHsp18.0-CI* was 10.6-, 13.1-, and 5.9- fold higher in OE3, OE10 and OE13 than in WT, respectively. Previous studies demonstrated that pathogenesis-related (*PR*) genes are induced in *OsHsp18.0-CI* overexpression lines without inoculation with RS105 (Ju et al. 2017). Then, three pathogenesis-related genes were measured with qRT-PCR to determine the basal levels of their transcripts in the *OsHsfB4d*-OE lines. All *PR* genes were increased expression in three OE lines compared with the WT. As shown in Fig. 4b-d, both *OsPR1a* and *OsPR1b* were significantly upregulated, and *OsPAL1* was slightly upregulated in one of the OE lines (OE10) compared to the WT. In addition, the increased levels of *PR* genes coincided with the increased level of *OsHsp18.0-CI* expression and the resistance performance of the three OE lines. These results indicated that the OE lines may enhance BLS resistance by activating the expression of *OsHsp18.0-CI* and *PR* genes.

### Knockout of OsHsfB4d has no Effect on Resistance to BLS and BB

**Fig. 5:**
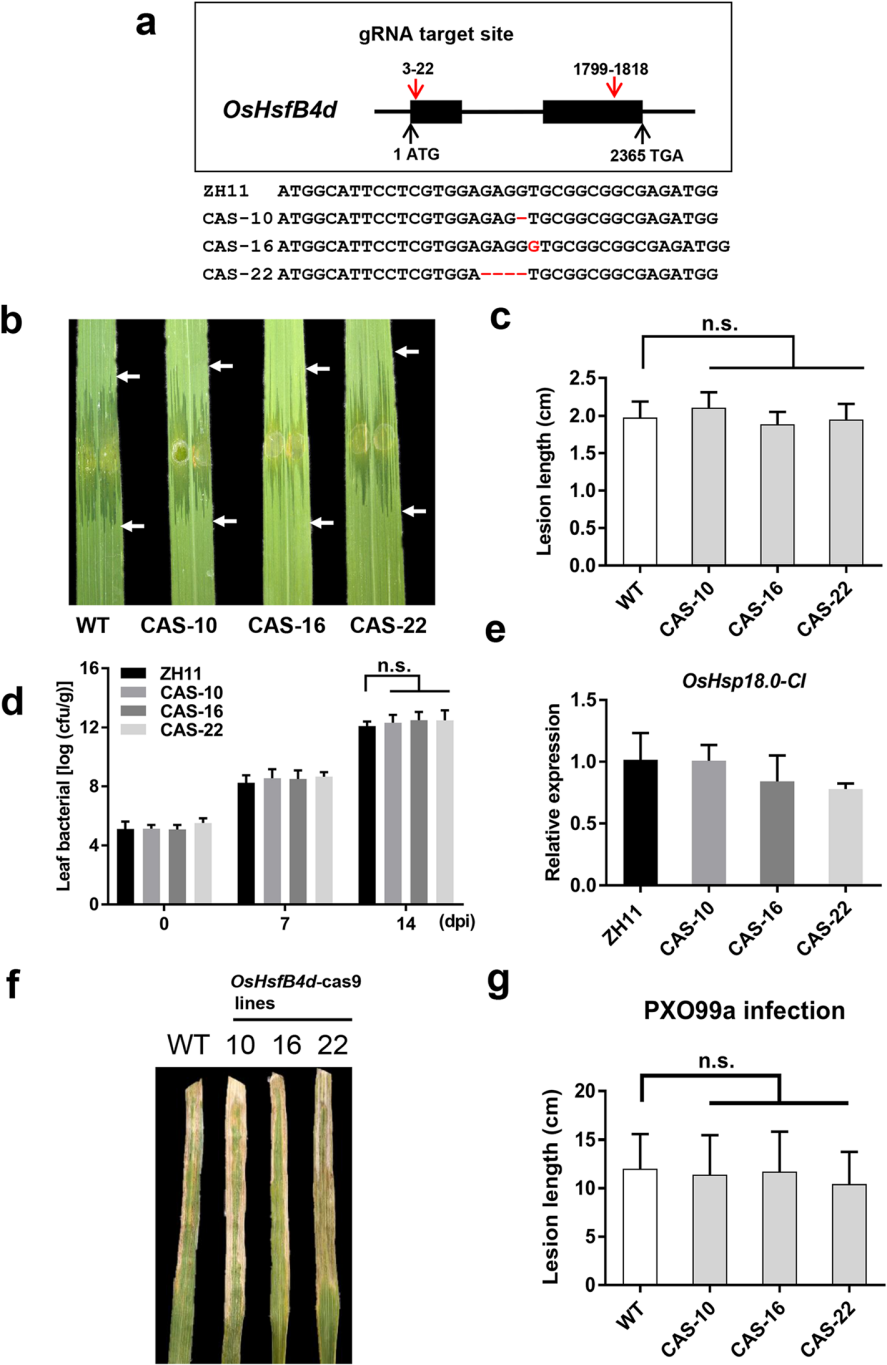
Resistance performance of transgenic *OsHsfB4d*-cas9 plants from the T_2_ generation. **a** Diagram of the Cas9 enzyme cleavage site and the genome editing of OsHsfB4d. Three mutation types, including a 1 base deletion, a 1 base insertion and a 4 bases deletion, were detected in the three transgenic lines. **b** Representative lesion sites from OsHsfB4d-cas9 plants at 14 dpi with Xoc strain RS105. **c** Lesion lengths in the OsHsfB4d-cas9 lines. Each line comprises seven individual transgenic plants assessed in this experiment at 6-week-old stage. “n.s.” means not significant according to the statistical analysis (n = 10). **d** Bacterial population growth in leaves of OsHsfB4d-cas9 and WT plants inoculated with RS105. The bars represent the mean ± SD. “n.s.”: not significant. **e** The transcript detection of *OsHsp18.0-CI* in 6-week-old wild-type and OsHsfB4d-cas9 lines. The bars represent the mean ± SD. **f** The phenotypes of the PXO99a lesions that developed on ZH11 and OsHsfB4d-cas9 lines at 14 dpi. **g** Lesion lengths were scored for OsHsfB4d-cas9 lines (n = 10). “n.s.”: not significant

To further identify the role of *OsHsfB4d* in rice-Xoc interactions, we generated CRISPR-Cas9 *OsHsfB4d* knockout lines in ZH11. Three independent individuals, CAS10, CAS16 and CAS22, were identified to have different null mutations and displayed various indel polymorphisms (Fig. 5a, Fig. S2). The CAS lines were assessed the resistance to RS105 in the T_2_ generations. At 14 dpi, the three knockout lines exhibited lesion lengths of 1.89 ± 0.16 cm, 1.95 ± 0.21 cm and 2.11 ± 0.21 cm, respectively, which were similar to the average of 1.98 ± 0.21 cm for WT plants (Fig. 5b, c). Consistent with these results, the bacterial populations in leaves were essentially unchanged between the CAS lines and WT at 7 dpi or 14 dpi (Fig. 5d). Additionally, the expression of *OsHsp18.0-CI* in the CAS lines were not significantly different from that in the WT (Fig. 5e).

By the BB inoculation, the lesion lengths between three knockout lines (CAS10, CAS16 and CAS22) and WT also did not changed significantly (Fig. 5f), the statistical lesion lengths were 11.4 ± 4.03 cm, 11.73 ± 4.08 cm, 10.43 ± 3.32 cm, 12 ± 3.55 cm, respectively (Fig. 5g). Overall, we concluded that the deletion of *OsHsfB4d* had little effect on the rice-*Xanthomonas* interaction.

### OsHsfB4d Directly Targets the Promoter of *OsHsp18.0-CI*

**Fig. 6:**
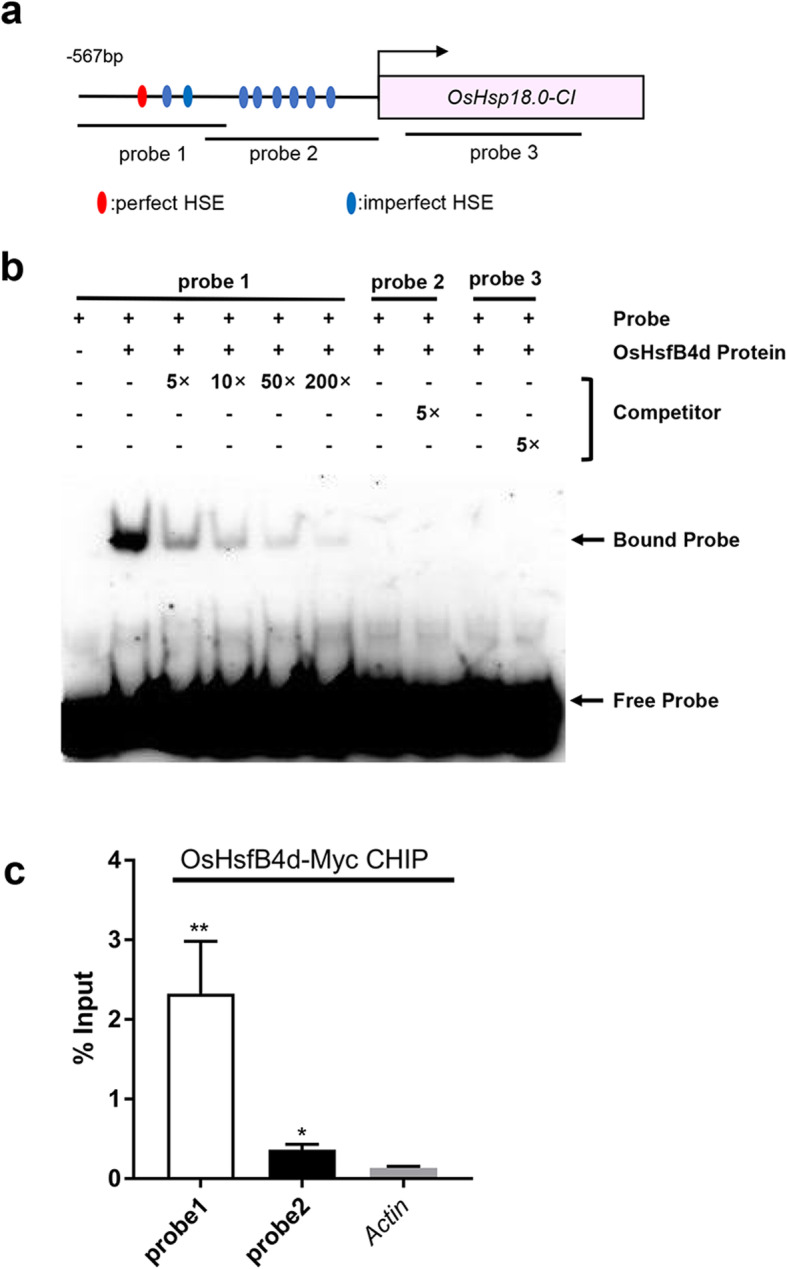
OsHsfB4d directly binds to the promoter of *OsHsp18.0-CI*. **a** Diagram of the positions of the primers and probes used in the EMSA and ChIP-qPCR experiments. **b** EMSA using recombinant His-tagged OsHsfB4d and the various deletion derivatives of the *OsHsp18.0-CI* promoter fragment. Lane 1 contained only the free probe. Lanes 2–7 contained the purified His-tagged OsHsfB4d and the probe. For the competitive EMSA, the purified OsHsfB4d protein was preincubated with 5×, 10×, 50×, 200× molar excess of unlabeled probe 1 (lane 3–6), 5× probe 2 (lane 8) and 5× probe 3 fragments (lane 10) before the addition of the corresponding biotin-labelled promoter fragment. **c** Binding of OsHsfB4d to the perfect HSE *cis*-element-containing fragment in a ChIP-qPCR assay. ChIP and EMSA experiment used the same primer pairs. The *Actin* was used as a nonspecific target gene

Hsfs usually regulate the expression of Hsp genes by directly targeting the HSE element to mediate physiological processes (Scharf et al. 2012). Nine putative HSEs, including one perfect HSE and eight imperfect HSEs, were previously predicted to be located in a 567 bp DNA fragment of the promoter of *OsHsp18.0-CI* by sequence analysis (Guan et al. 2004). To investigate the interaction between OsHsfB4d and the *OsHsp18.0-CI* promoter, a His-tagged recombinant OsHsfB4d protein was purified via prokaryotic expression and subsequently used to assess binding ability with electrophoretic mobility shift assays (EMSA). The results showed that OsHsfB4d could bind to DNA probe 1, which contained the perfect HSE motif but failed to bind DNA probe 2 and probe 3, which are located at other positions in *OsHsp18.0-CI* (Fig. 6a, b, Fig. S3). To further confirm the binding ability, we performed ChIP-qPCR assays using OE10 rice plants carrying OsHsfB4d-Myc. Consistent with the EMSA results, relatively greater enrichment of the probe 1 DNA fragment, containing the perfect HSE motif, was observed during immunoprecipitation than that of the DNA fragment of probe 2 or *β-actin* by using anti-Myc agarose beads (Fig. 6c). Based on the results of EMSA and ChIP-qPCR, we concluded that OsHsfB4d directly binds to the *OsHsp18.0-CI* promoter.

**Fig. 7:**
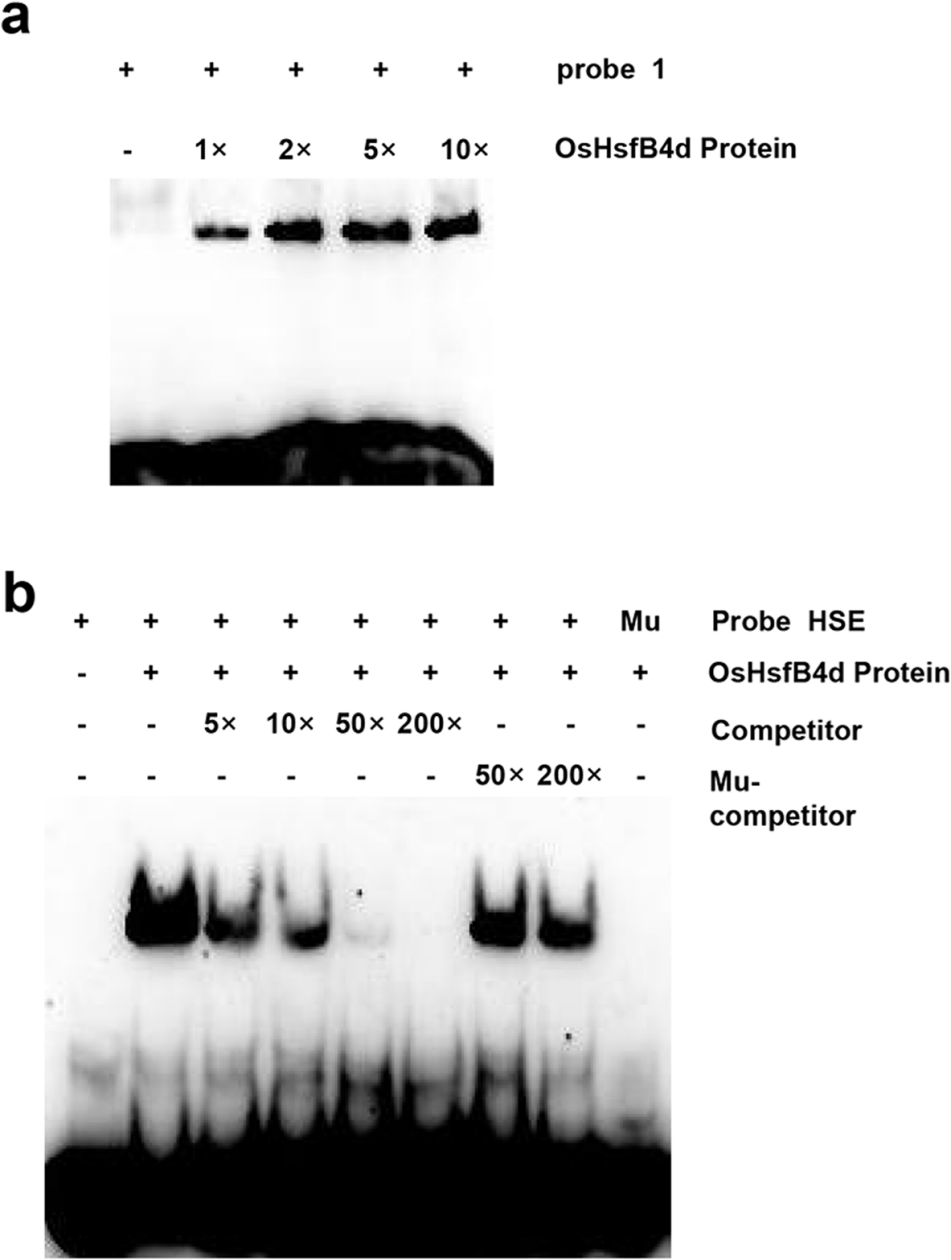
OsHsfB4d binds to the perfect HSE motif with the *OsHsp18.0-CI* promoter. **a** EMSA using the probe 1 and different amount of recombinant His-tagged OsHsfB4d. 1× represents about 0.53 μg of the purified OsHsfB4d. **b** OsHsfB4d bind to the perfect HSE *cis*-element. The OsHsfB4d protein was incubated with the labeled probe to serve as a positive control; mutated probes (Mu) were used as a negative control. 5-, 10-, 50- and 200-fold excesses of unlabeled probes were used for competition. 50- and 200-fold excesses of mutated probes were used for competition. Mutated probe in which the perfect HSE motif 5′- GAAACTTC − 3′ was replaced with 5′- CAAACAAT -3′

Considering the probe 1 DNA fragment bound to OsHsfB4d contains a perfect HSE motif and two imperfect ones, we further tested the interaction with shorter fragment containing the only perfect HSE motif. Firstly we fixed amount of labeled probes and uploaded different amount of proteins to detect the effect of the protein concentration on the protein-DNA interaction. The result showed the increase of bound probes with the increased amount of proteins (Fig. 7a), further confirmed the binding of probe 1 DNA fragment to OsHsfB4d. EMSA image demonstrated that OsHsfB4d protein bound the DNA probe containing the perfect HSE motif but failed to bind the similar DNA probe in which the HSE motif was mutated (Fig. 7b), indicating that OsHsfB4d specifically binds the perfect HSE motif of *OsHsp18.0-CI* promoter.

## Discussion

Class B Hsfs are generally regarded as negative regulators that respond to abiotic stresses in plants. AtHsfB1 and AtHsfB2b have been shown to repress the induction of *Hsps* during stress recovery in *Arabidopsis* (Ikeda et al. 2011). Soybean HsfB2b inhibited *GmNAC2* expression by directly binding to the HSE element in the salt stress response (Bian et al. 2020). To date, there have been few reports on the involvement of class B Hsfs in regulating the plant response to biotic stress. Furthermore, *AtHsfB1* and *AtHsfB2b* have been shown to negatively regulate resistance to *A. brassicicola* by suppressing the expression of the plant defensin genes *PDF1.2a* and *PDF1.2b* (Kumar et al. 2009; Liu and Charng 2013). Do class B Hsfs act as positive regulators to directly activate the expression of targeted genes? In tomato, *SlHsfB1* alone represses the transcription of *Hsps*, whereas it can positively regulate the transcription of *Hsps* by forming heterodimers with SlHsfA1 (Hahn et al. 2011). In addition to inhibiting *GmNAC2* expression, HSFB2b also plays as a positive regulator that can bind to the promoter and activate the expression of *GmC4H* to promote flavonoid biosynthesis in response to salt stress in soybean (Bian et al. 2020). Here we also revealed that one of the class B Hsfs, *OsHsfB4d*, activates the expression of the target gene *OsHsp18.0-CI* (Fig. 4a) and enhances BLS and BB resistance (Fig. 3b-h) after overexpression in rice. Thus, we revealed a new scenario in which class B Hsfs may also act as positive regulators in response to biotic stresses.

In contrast to class A Hsfs, class B and C Hsfs lack the activation domain, so they are not complete transcription factors (Czarnecka-Verner et al. 2000, 2004; Kotak et al. 2004; Lavania et al. 2018). Previous studies demonstrated that the Hsfs of subclass A act as master regulators of the heat stress response (Mishra et al. 2002; Liu et al. 2011; Yoshida et al. 2011). Class B Hsfs usually play roles as co-activators or co-repressors (Czarnecka-Verner et al. 2000, 2004; Bharti et al. 2004; Ikeda et al. 2011; Schmidt et al. 2012). In this study, although we found that OsHsfB4d could directly bind to the promoter as well as activate the expression of *OsHsp18.0-CI* or *GFP* in *OsHsfB4d* OE lines (Fig. 4a) or in a co-transient expression system (Fig. 2b-d), it is still unclear whether OsHsfB4d could independently activate the expression of *OsHsp18.0-CI*. Because OsHsfB4d also lacks an activation domain and transactivation activity in yeast (Mittal et al. 2011; Lavania et al. 2018), we suggest that OsHsfB4d may act as a necessary cofactor in the transcriptional regulation of *OsHsp18.0-CI*, and the key regulator is conserved and exists in both rice and *N. benthamiana*.

There are 25 typical *Hsfs* genes in the rice genome. Of these, 13, 8 and 4 Hsfs belong to class A, class B and class C, respectively (Chauhan et al. 2011). We analysed all the transcription data for class A and class B *Hsfs* in the *OsHsp18.0-CI* OE lines post-inoculation with Xoc or Xoo. *Hsfs* exhibited various expression patterns in transgenic plants or after inoculation with different pathogens (Fig. S1), implying that they play different roles in the immune responses of rice. In this study, overexpression of *OsHsfB4d* enhanced resistance to the Xoc and Xoo (Fig. 3b-h), suggesting that *OsHsfB4d* positively mediates immunity in rice. Surprisingly, the three *OsHsfB4d* knockout lines exhibited no difference in BLS and BB resistance (Fig. 5b-g), implying that there are redundant activators that regulate the expression of *OsHsp18.0-CI.* Interestingly, the 567 bp-long promoter of *OsHsp18.0-CI* contains nine putative *cis*-elements of HSE, the only one perfect HSE element was confirmed to precisely bind to the *OsHsp18.0-CI* promoter (Fig. 7b) It would be available to be bound via additional HSE sites by other OsHsfs that need to cooperate with OsHsfB4d. Furthermore, this is a bidirectional promoter that drives the expression of *OsHsp17.3* in an alternative orientation. The expression of *OsHsp17.3* was induced in response to L-azetidine-2-carboxylic acid (AZC) and heat stress in rice and was coexpressed with *OsHsfA4b* in *N.benthamiana* (Guan et al. 2010). Therefore, the specific redundant activator of OsHsfB4d should be further investigated.

*OsHsp18.0-CI* has been identified to respond to treatment with anoxia, heat, cold, salt, drought, AZC, cadmium, and the bacterial pathogens Xoo and Xoc (Guan et al. 2004; Sarkar et al. 2009; Cui et al. 2013; Ham et al. 2013; Ju et al. 2017; Zuo et al. 2019). Several studies have indicated that overexpression of *OsHsp18.0-CI* enhances tolerance to abiotic and biotic stresses (Ham et al. 2013; Ju et al. 2017; Zuo et al. 2019). As *OsHsfB4d* is the upstream regulator of *OsHsp18.0-CI*, we showed that *OsHsfB4d* overexpression in rice enhanced BLS and BB resistance. It would be very interesting to expand the assessment to include more abiotic and biotic stressors in the future. OsHsfB4d maybe a good choice for breeding better rice varieties that are adapted to complex stress conditions in changed environments.

In conclusion, we showed that OsHsfB4d is a positive regulator involved in the rice-Xoc and rice-Xoo interaction. It could directly bind to the promoter and regulate the expression of *OsHSP18.0-CI* to enhance BLS and BB resistance in rice.

## Methods

### Plant Cultivation and Pathogen Inoculation

Seeds of the rice variety ZH11 and the transgenic lines were germinated and grown in a greenhouse at 28 ± 2 °C with 80% relative humidity and a 12-h photoperiod. The *N.benthamiana* plants used in this experiment were cultured in a chamber at 25 °C with a 16 h light / 8 h dark cycle. For inoculation, the Xoc strain RS105 and Xoo strain PXO99a was grown on PSA medium at 28 °C for 2 d and then suspended in 10 mM sterile MgCl_2_ at an OD600 = 0.5. For Xoc inoculation, at least five fully expanded leaves of 6-week-old plants were infiltrated at three positions by inoculation with a needleless syringe (Ju et al. 2017). The lengths of the lesions on the transgenic and wild type plants were scored at 14 dpi. For Xoo inoculation, 6-week-old rice plants were inoculated with PXO99a by the leaf-clipping method (Yang et al. 2016) and lesion lengths were measured at 14 dpi.

### Vector Construction and Rice Transformation

The cDNAs of the LOC_Os03g25120 (*OsHsfB4d*) genes were amplified by RT-PCR using specific primers (Table S1). The *OsHsfB4d* fragment was cloned into the pCXUN-Myc vector under the control of the ubiquitin promoter (Chen et al. 2009). The CRISPR-OsHsfB4d vectors were generated according to the instructions (Ma et al. 2015). In brief, two sites for guide RNA (gRNA) targeted to the exon of OsHsfB4d were designed and transcribed from the U3 and U6a promoters, respectively, and subsequently cloned into the pYLCRISPR/Cas9-MH binary vectors. Then, the constructs were transferred into the *Agrobacterium tumefaciens* strain EHA105. All the constructs mentioned above were introduced into ZH11 using the standard *Agrobacterium*-mediated transformation system described previously (Li et al. 2013).

### RNA Extraction and Real-Time RT-PCR

Pathogen-infected and control plant leaves were harvested at 24 h posttreatment for RNA isolation by using a Plant RNA Extraction Kit (Omega Bio-Tek, USA). First-strand cDNA was generated using the ReverTra Ace qPCR RT Master Mix with gDNA Remover kit (TOYOBO, Japan). Quantitative real-time PCR was performed on a qTOWER^3^G touch (Analytikjena, Germany) with KOD SYBR qPCR Mix (TOYOBO). The PCR program was performed as described in a previous study (Yang et al. 2019). The gene expression levels relative to those of the rice *OsActin* (LOC_Os03g50890) gene were analysed using the 2^-△△Ct^ analysis method. The *OsActin* gene was used as an internal control to standardize the results. Gene expression levels were analysed by qRT-PCR assays, which were repeated at least twice in triplicate.

### Fluorescence Microscopy Assay

Green fluorescent protein (GFP) was used as a reporter to investigate the expression of target gene fragments *in planta*. The pCXGFP-OsHsfB4d constructs were introduced into *A. tumefaciens* strain GV3101 and transiently expressed in *N. benthamiana* epidermis cells. GFP fluorescence was observed under a Leica M205 C stereomicroscope as described previously (Li et al. 2017; Yang et al. 2017). The fluorescence was quantified using an EnSpire Multimode Plate Reader (PerkinElmer, USA) as described previously (Yang et al. 2017).

### Luciferase Reporter Assay

The constructs were transformed into *Agrobacterium* strain GV3101. Overnight cultures were collected by centrifugation, resuspended in MES buffer (10 mM MES pH 5.6, 10 mM MgCl_2_, and 150 mM acetosyringone) at an OD600 of 0.5 and incubated at room temperature for 2–3 h. The suspension was infiltrated into healthy leaves of 3-week-old *N. benthamiana* plants with a 2 mL needleless syringe. The plants were left under normal condition in chamber for 2 d after infiltration. Luciferin (1 mM) was infiltrated before the LUC signal was photographed with Night Shade LB985 (Berthold Technologies, Germany). The primer sequences used in this study are listed in Table S1.

### EMSA

Full-length CDSs of *OsHsfB4d* were amplified and cloned into pET-28a.The recombinant 6 × His fusion proteins were expressed in *E. coli* BL21 (DE3) and purified to homogeneity using a Ni-NTA (Roche, USA) resin column. The oligonucleotide probes were amplified by the EMSA primers listed in Table S1 and labelled with biotin at the N′ end of the PCR product by the EMSA Probe Biotin Labelling Kit (Beyotime, China). The oligonucleotides containing only perfect elements were synthesized by Sangon Biotech (China) with 5′-biotin marked. EMSAs were performed as previously described (Li et al. 2018). Briefly, biotin-labelled probes were incubated with His-tagged OsHsfB4d protein at room temperature for 20 min, and the subsequent steps were performed using a Chemiluminescent EMSA Kit (Beyotime, China) according to the manufacturer’s instructions. Free and bound probes were separated via PAGE, transferred to nylon membranes and subjected to chemiluminescence examination.

### ChIP-qPCR Assay

Leaves of 3-week-old pCXUN-Myc-OsHsfB4d seedlings were collected. Approximately 1 g leaves was harvested and cross-linked in 1% (v/v) formaldehyde at room temperature for 10 min, followed by neutralization with 0.125 M glycine. The chromatin-protein complex was isolated, resuspended in lysis buffer (50 mM Tris, 150 mM NaCl, 1 mM EDTA, 1% (w/v) SDS, 1% (v/v) Triton X-100, 1 mM PMSF, and 1% protease inhibitor mixture), and sheared by sonication to reduce the average DNA fragment size to 500 bp. Then, 50 μL of sheared chromatin was saved for use as an input control. ChIP was conducted using a ProFound™ c-Myc Tag IP/Co-IP kit according to the manufacturer’s instructions (Thermo Scientific, USA). The protein-DNA cross-links were reversed by incubating the immunoprecipitated complexes with 20 μL of 5 M NaCl at 65 °C overnight. The DNA was recovered by ethanol precipitation, washed with cold 75% ethanol and dried. The pellet was dissolved in water and analysed by qPCR. The enrichment of the target gene promoters is shown as the percentage of the input DNA. Actin was used as a nonspecific target gene. The primers used for qPCR are listed in Table S1.

### Western-Blot Assay

Approximately 1 g of fresh rice leaves was ground in liquid nitrogen, resuspended in extraction buffer (50-mM Tris-HCl, pH 7.4, 150-mM NaCl, 5-mM EDTA, 1-mM PMSF, 1% NP-40 and 1× protease inhibitor cocktail) and then centrifuged at 12,000 g for 15 min at 4 °C. An aliquot of 100 μL of supernatant was boiled for 5 min with 1× SDS buffer. The protein samples were separated by SDS-PAGE using a 10% gel. Proteins were blotted onto a PVDF transfer membrane (Millipore, USA). OsHsfB4d protein was detected by a monoclonal anti-Myc antibody (Abcam, USA). The images were photographed using SuperSignal® West Dura Extended Duration Substrate (Thermo Scientific). The stained Rubisco protein shows the equal loading of protein samples.

### Data Treatment

The accession number of the original transcription data of *OsHsp18.0-CI* is SRP079496 (Ju et al. 2017; Zuo et al. 2019). Quantitative data were analysed using Student’s *t* test (two-tailed *t* test with equal variances; Microsoft Excel) to evaluate the significance of the differences between wild-type plants and other plants. *Indicates significant (*t* test, *P* < 0.05) differences and **indicates *P* < 0.01.

## Supplementary information

**Additional file 1: Table S1.** The primers used in this study.

**Additional file 2: Fig. S1.** Heatmaps showing the expression patterns of class A and B Hsfs in *Hsp18.0-CI* OE and WT plants post inoculation with Xoo or Xoc.

**Additional file 3: Fig. S2.** Sequencing result for the fragment around the PAM in the wild type and the three OsHsfB4d-cas9 lines.

**Additional file 4 Fig. S3** Nucleotide sequence of the promoter region of *OsHsp18.0-CI*. The translation start site of *OsHsp18.0-CI* is underlined with dots. The perfect HSE element is marked with light gray and the eight imperfect HSE are shown by dark gray. The probe 1 sequence is underlined in bold and the probe 2 sequence is indicated with wavy line, there is an overlap between the two sequences. Synthetic probe with only perfect HSE element is indicated by bold italic letters.

## Data Availability

The data that support the findings of this study are available from the corresponding author on reasonable request.

## Abbreviations

BB: Bacterial blight
BLS: Bacterial leaf streak
DEG: Differentially expressed genes
dpi: Days post inoculation
DR: Defense-related
GFP: Green fluorescent protein
Hsfs: Heat shock factors
HSPs: Heat shock proteins
Luc: Luciferase
OE: Overexpression
PR: Pathogenesis related
QTLs: Quantitative trait loci
WT: Wild-type
Xoc: *Xanthomons oryzae* pv. *oryzicola*
Xoo: *Xanthomonas oryzae* pv. *oryzae*
