# Supplementary material for: OsHsfB4d Binds the Promoter and Regulates the Expression of OsHsp18.0-CI to Resistant Against Xanthomonas Oryzae
Source: Rice (N Y). 2020 May 27;13:28. doi: 10.1186/s12284-020-00388-2 (PMC7253548; doi:10.1186/s12284-020-00388-2)
Supplement: Supplementary file 1 — Additional file 1: Table S1. The primers used in this study. [file 12284_2020_388_MOESM1_ESM.docx]

**Table S1.** The primers used in this study

| **Name** | **Sequence (5'-3')** |
| --- | --- |
| ***Ubi-P-SEQ*** | **TTTTAGCCCTGCCTTCATACGC** |
| ***35S-SEQ*** | **GCAAGTGGATTGATGTGATATC** |
| ***NosR-SEQ*** | **AGACCGGCAACAGGATTCAATC** |
| ***HSFB4D-CDS-F*** | **ATGGCATTCCTCGTGGAGAGGTG** |
| ***HSFB4D-CDS-R*** | **CTACCCCTCGCTTCCCTGCTCGT** |
| ***HSFB4D-U3-gRNA-F*** | **ggcAGCATTCCTCGTGGAGAGGTG** |
| ***HSFB4D-U3-gRNA-R*** | **aaacCACCTCTCCACGAGGAATGC** |
| ***HSFB4D-U6a-gRNA-F*** | **gccGATCTCCGAGAGGAGGTGCT** |
| ***HSFB4D-U6a-gRNA-R*** | **aaacAGCACCTCCTCTCGGAGATC** |
| ***gateway-attB-HSFB4D-F*** | **GGGGACAAGTTTGTACAAAAAAGCAGGCTTCATGGCAT**  **TCCTCGTGGAGAGGTG** |
| ***gateway-attB-HSFB4D-R*** | **GGGGACCACTTTGTACAAGAAAGCTGGGTCCTACCCCT**  **CGCTTCCCTGCTCGT** |
| ***YW-HSP18.0-LUC-KpnI-F*** | **GCGGTACCgctagatatattaatatat** |
| ***YW-HSP18.0-LUC-BamHI-R*** | **GCGGATCCTGTGTATTGTGTCTTGCTG** |
| ***YW-HSP18.0-Promoter-GFP-F*** | **cgtcggaatagctgcgaatttgg** |
| ***YW-HSP18.0-Promoter-GFP-R*** | **TGTGTATTGTGTCTTGCTG** |
| ***QRT-18.0-F*** | **GGTGGAGAGCTTCGATTCGA** |
| ***QRT-18.0-R*** | **GGACCAGATTTGACGCTTTTATTT** |
| ***QRT-B4D-F*** | **GCACATGAGGAAGCTCTACAAC** |
| ***QRT-B4D-R*** | **CTTCCCTGCTCGTCTCCTT** |
| ***QRT-PR1a-F*** | **CGTCTTCATCACCTGCAACTACTC** |
| ***QRT-PR1a-R*** | **CATGCATAAACACGTAGCATAGCA** |
| ***QRT-PR1b-F*** | **GGCAACTTCGTCGGACAGA** |
| ***QRT-PR1b-R*** | **CCGTGGACCTGTTTACATTTTCA** |
| ***QRT-PAL1-F*** | **AGCACATCTTGGAGGGAAGCT** |
| ***QRT-PAL1-R*** | **GCGCGGATAACCTCAATTTG** |
| ***QRT-OsActin-F*** | **TGTATGCCAGTGGTCGTACCA** |
| ***QRT-OsActin-R*** | **CCAGCAAGGTCGAGACGAA** |
| ***18.0-Promoter-EMSA-1F*** | **cgtcggaatagctgcgaatttgg** |
| ***18.0-Promoter-EMSA-1R*** | **ctggacgctatgggctacagcc** |
| ***18.0-Promoter-EMSA-2F*** | **ggctgtagcccatagcgtccag** |
| ***18.0-Promoter-EMSA-2R*** | **tgtgtattgtgtcttgctgtttt** |
| ***18.0-Promoter-EMSA-3F*** | **cccttcgacggcttccccttcggct** |
| ***18.0-Promoter-EMSA-3R*** | **atctggaggacgttgccgtcctc** |
| ***18.0-Promoter-chip-qRT-1F*** | **CGTCGGAATAGCTGCGAATTTGG** |
| ***18.0-Promoter-chip-qRT-1R*** | **CTGGACGCTATGGGCTACAGCC** |
| ***18.0-Promoter-chip-qRT-2F*** | **GGCTGTAGCCCATAGCGTCCAG** |
| ***18.0-Promoter-chip-qRT-2R*** | **TGTGTATTGTGTCTTGCTGTTTT** |
| ***18.0-HSE-biotin-F*** | **biotin-cttggaataagcttctagaaacttccagtttg** |
| ***18.0-HSE-F*** | **cttggaataagcttctagaaacttccagtttg** |
| ***18.0-HSE-mutant-biotin-F*** | **biotin-cttggaataagcttctacaaacaatcagtttg** |
| ***18.0-HSE-mutant-F*** | **cttggaataagcttctacaaacaatcagtttg** |
| ***18.0-HSE-R*** | **CAAACTGGAAGTTTCTAGAAGCTTATTCCAAG** |
| ***18.0-HSE-mutant-R*** | **CAAACTGATTGTTTGTAGAAGCTTATTCCAAG** |
