# Supplementary figures and images for: OsHsfB4d Binds the Promoter and Regulates the Expression of OsHsp18.0-CI to Resistant Against Xanthomonas Oryzae
Source: Rice (N Y). 2020 May 27;13:28. doi: 10.1186/s12284-020-00388-2 (PMC7253548; doi:10.1186/s12284-020-00388-2)

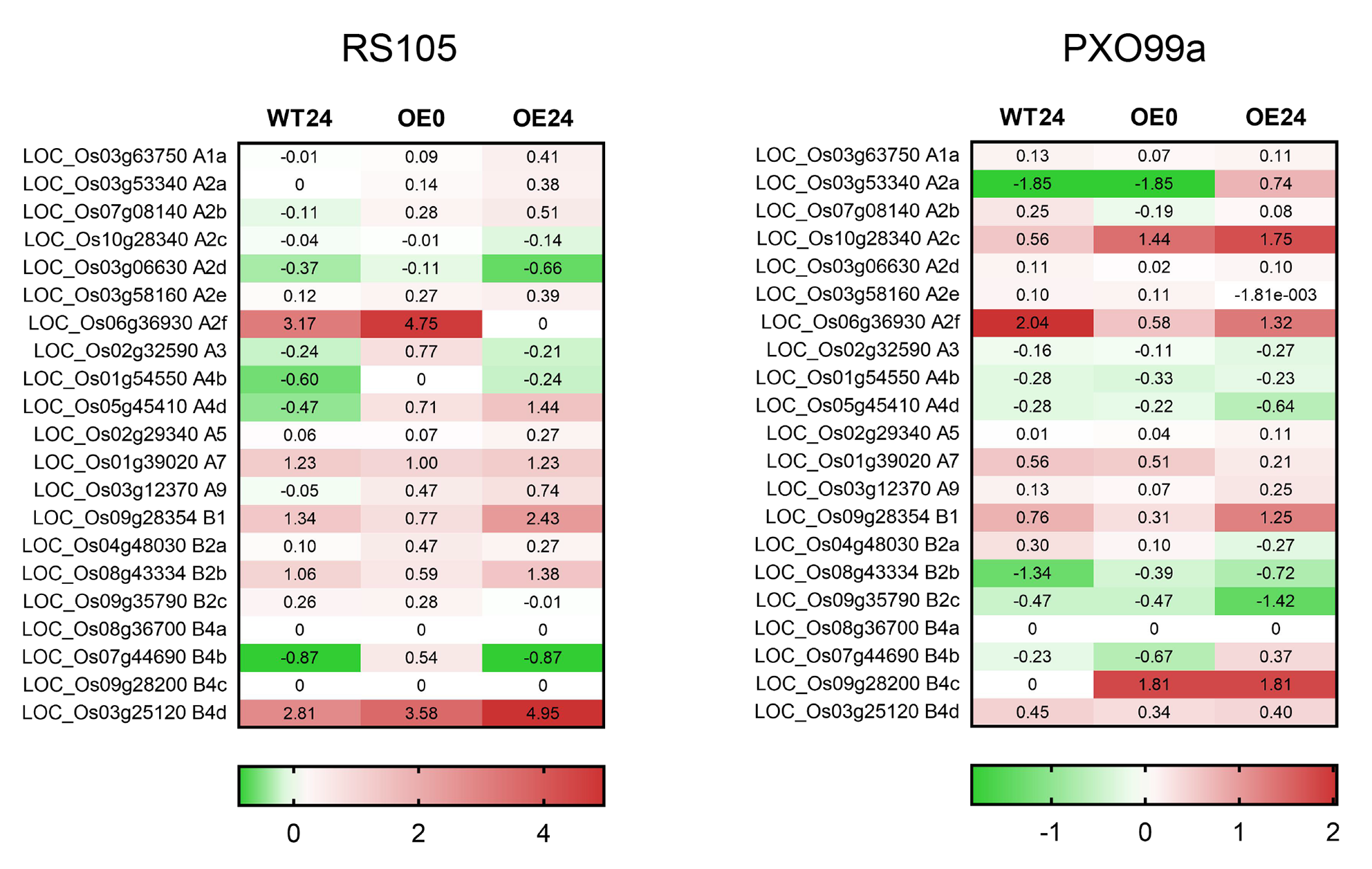

Supplement: Supplementary file 2 — Additional file 2: Fig. S1. Heatmaps showing the expression patterns of class A and B Hsfs in Hsp18.0-CI OE and WT plants post inoculation with Xoo or Xoc. [file 12284_2020_388_MOESM2_ESM.tif]

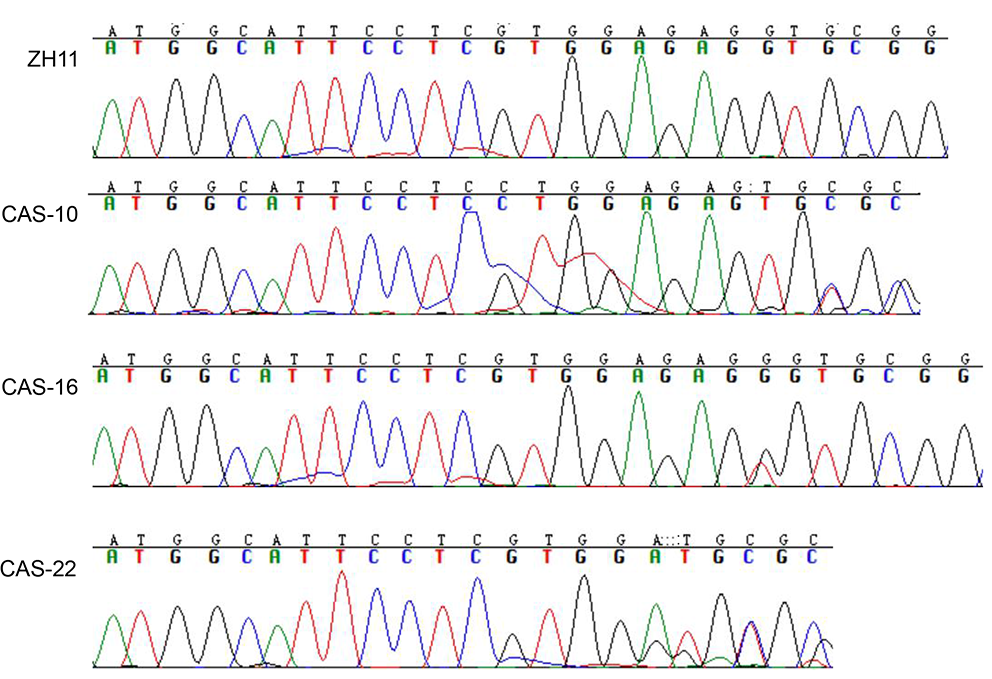

Supplement: Supplementary file 3 — Additional file 3: Fig. S2. Sequencing result for the fragment around the PAM in the wild type and the three OsHsfB4d-cas9 lines. [file 12284_2020_388_MOESM3_ESM.tif]

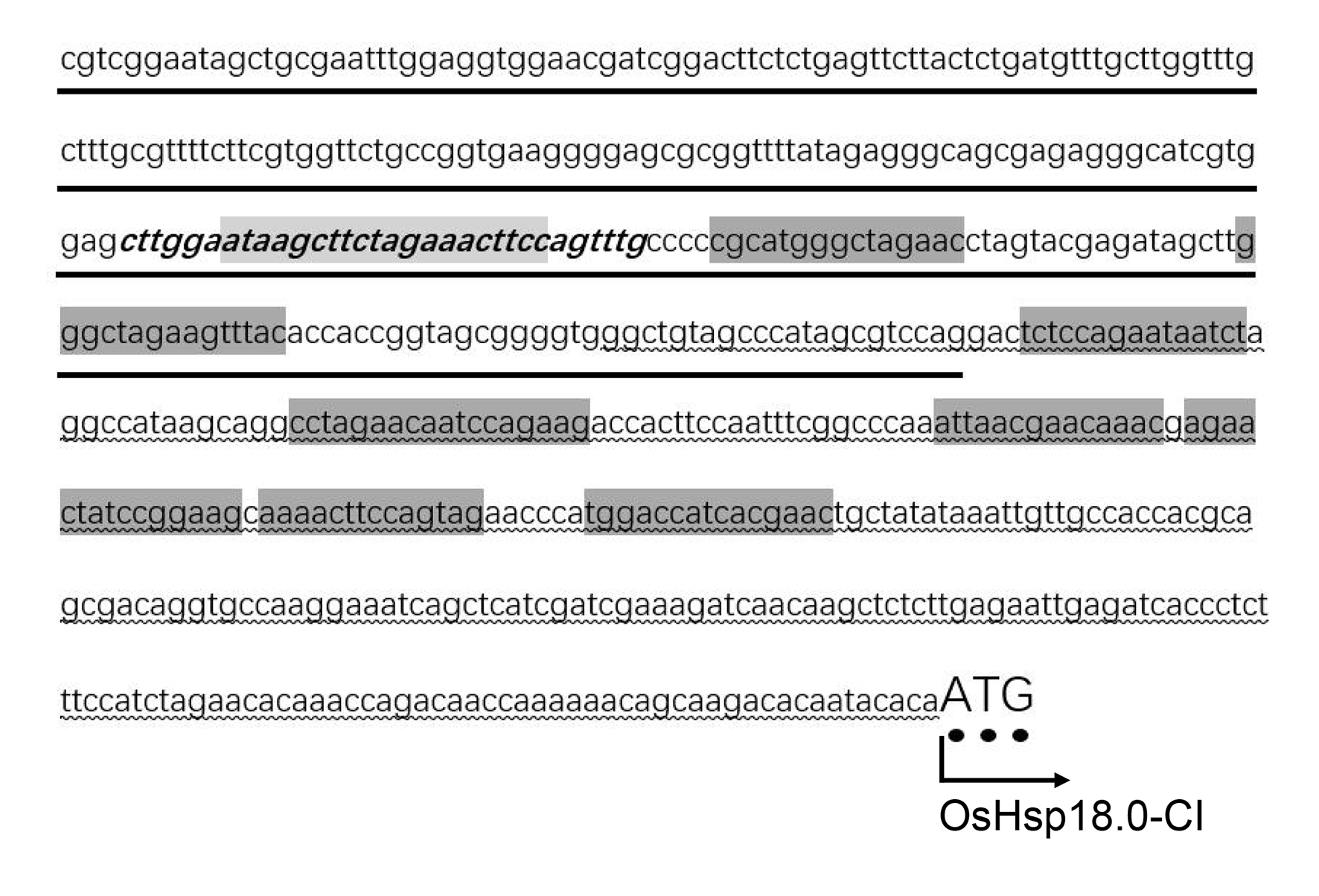

Supplement: Supplementary file 4 — Additional file 4 Fig. S3 Nucleotide sequence of the promoter region of OsHsp18.0-CI. The translation start site of OsHsp18.0-CI is underlined with dots. The perfect HSE element is marked with light gray and the eight imperfect HSE are shown by dark gray. The probe 1 sequence is underlined in bold and the probe 2 sequence is indicated with wavy line, there is an overlap between the two sequences. Synthetic probe with only perfect HSE element is indicated by bold italic letters. [file 12284_2020_388_MOESM4_ESM.tif]
